# On Superfœtation

**Published:** 1810-06

**Authors:** 


### To the Editors of the Medical and Phyfical Journal.

GENTLEMEN,

IN the number of your Journal for this month, a curious case of twins is communicated, by an unknown Correspondent, as introductory to a new doctrine of superfœtation. It would appear, however, according to the Correspondent's own apprehensions, that circumstances are not at present the most favourable for the reception of a new doctrine; inasmuch as the ancient, and hitherto *only* doctrine of superfœtation, is so far from having been given up, that he says, "the majority of medical practitioners have, by no means, made up their minds on the subject." The advocates of the ancient, and I repeat it, hitherto *only* doctrine of superfœtation, cannot but charge the Correspondent with some inconsistency, in affirming, after having said, that *above half* of the medical practitioners have not made up their minds upon it, that "*no person* who has attentively considered the anatomy of the gravid uterus, can for a moment suppose it" [the ancient doctrine] to possess any truth. This affirmation is the more extraordinary, because it is certain, that *one person* at least, Mr. Fawell, of Leeds, has declared, not long back, in your Journal, his faith in the old doctrine, and has recorded what he calls, "a Case of Superfœtation" in support of it. Mr. Fawell's case, which is similar in all its circumstances to that published in your last number, was ingeniously animadverted on by one, who, although he made some *Concessions*, could not admit the doctrine of superfœtation. Now it is very remarkable, that these *concessions* strictly coincide with that *doctrine* announced in your last number, as a novelty which had not "as yet been published by any author on the subject." That the Correspondent, whose paper is signed with an H. may not suppose that I am dealing unfairly with him, I here give reference to the numbers of your Journal, containing the subjects in question. No. 125, p. 47, and No. 128, p. 293. But regarding the correctness of the Correspondent's views of superfœtation, I have to observe, that they must appear to a great part of the medical men who consider the notion of superfœtation as unphilosophical, [and I confess myself one of that sort] to be founded on little more than the inconclusiveness of his resoning against the doctrine of our ancestors. The ancients believed that a parent, when several months gone in pregnancy, might, before the birth of the offspring, conceive again. The amount of his objections is, that when pregnancy is so far advanced, the os uteri is plugged up by a tough gelatinous substance, and the ovaria in consequence inaccessible to the seminal fluid. The want of communication to the ovaria being set down as the only obstacle to superfœtation, it irresistibly follows that superfœtation *may* take place whenever this want of communication does not exist. Thus, while the os uteri is not impermeable, while nothing obstructs the passage from the vagina to the Fallopian tubes, as is the case "in the early stages of pregnancy," the already impregnated uterus admits of impregnation again. The sum and substance, then, of the doctrine is, that a pregnant animal remains *susceptible* of further impregnation during *the whole period of pregnancy*; for if no susceptibility exists, the stoppage of the os uteri cannot "*prevent*" impregnation: for to talk of *preventing* what cannot happen is to talk without meaning. As it is a gratuitous assumption, incongruous with the facts of gestation, that the female organs may remain susceptible of further impregnation while impregnated, all reasoning in support of the position must be found fallacious. The fact is, that the stoppage of the os uteri does not "*prevent*" impregnation, it can only prevent the passage of the seminal fluid to the Fallopian tubes; and the stoppage alone is not sufficient for that in every instance. If the "gelatinous substance" which plugs up the os uteri was in all cases "as unyielding as the hard substance of the uterus itself," a danger attends unassisted flooding cases, which has I believe never been expected,—*rupture of the uterus*.

Although we are ignorant why some animals bear only one at a time, and others many, this much I think is certain, that the doctrine of superfœtation is incompatible with *the fact*. When the process of fecundation is completed, [which, according to the experiments of Dr. Haighton takes up many hours] the sexual organs have undergone a change, which *prevents* further impregnation during the period of gestation. If the sexual organs were otherwise constituted, *all* animals would be multifarious, and multifarious too, beyond all determinate bounds.

The alleged examples of superfœtation occur chiefly in the lower order of animals, from their having admitted varieties of their species before the process of fecundation has terminated.

With regard to the black woman in America, spoken of by by your Correspondent, who had *a black child and a mulatto* at one lying-in, I do not think it was worth while to make a theory on her account, especially such an one as is better calculated to increase the difficulty of the fact than to solve it. The first step most assuredly was, as no two accounts agree, *to ascertain the fact.* In number 128 of your Journal, this woman is said to have had "a *white* and a black child at the same birth;" in the last Number the account is, that "one was a black child, and the other *a mulatto*;" and from the uncertainty in which the fact is involved, I will not presume to extricate it. Professor Lobstein, in his "Essai sur la nutrition du fœtus," has made out the strongest case of imputed superfœtation. It is the case of a woman with two uteri and two vaginæ,* who having been delivered of one child, was, in about a month afterwards delivered of another. The extraordinary part of the case is the *interval* between the birth of the children. The woman having two uteri affords no explanation; because in those animals which have double uteri, the actions of both uteri to expel their contents *are synchronous.*

With respect to the Correspondent's case of twins, in which the latter fœtus was still-born, and much less than the other, I need not say that I perfectly agree with him, that it was not a case of superfœtation. He is of opinion, that the still-born fœtus was killed by an accident, which the mother had when about five months gone with child. Of this, I think, much doubt exists. Be this as it may, the case is very interesting, and well deserving the consideration of your numerous readers.

I am, &c.

A. B.

*April* 11, 1810.

